# Efficacy and Safety of Intensity-Modulated Radiotherapy Following Transarterial Chemoembolization in Patients With Unresectable Hepatocellular Carcinoma

**DOI:** 10.1097/MD.0000000000003789

**Published:** 2016-05-27

**Authors:** Tao Zhang, Yu-Ting Zhao, Zhi Wang, Cheng-Rui Li, Jing Jin, Angela Y. Jia, Shu-Lian Wang, Yong-Wen Song, Yue-Ping Liu, Hua Ren, Hui Fang, Hui Bao, Xin-Fan Liu, Zi-Hao Yu, Ye-Xiong Li, Wei-Hu Wang

**Affiliations:** From the Departments of Radiation Oncology (TZ, Y-TZ, ZW, JJ, S-LW, Y-WS, Y-PL, HR, HF, X-FL, Z-HY, Y-XL, W-HW), Interventional Radiology (C-RL), Cancer Hospital, Chinese Academy of Medical Sciences (CAMS) and Peking Union Medical College (PUMC), Beijing, China; Department of Medicine (AYJ), Weill Cornell Medical College, New York City, NY; and Department of Oncology (HB), Yan’an University Affiliated Hospital, Yan’an Shaanxi Province, China.

## Abstract

Three-dimensional conformal radiotherapy in combination with transarterial chemoembolization (TACE) has been beneficial in patients with unresectable hepatocellular carcinoma (HCC). There have been few clinical reports on the use of intensity-modulated radiotherapy (IMRT) in combination with TACE for these patients. The purpose of this study was to assess the efficacy and toxicity of IMRT following TACE in unresectable HCC.

The medical records of consecutive patients with unresectable HCC, who underwent IMRT following TACE from January 2009 to June 2014, were retrospectively reviewed in order to assess the overall survival (OS), progression-free survival (PFS), tumor response, and treatment-associated toxicity.

A total of 64 lesions in 54 patients were included in the analysis. IMRT was delivered at a median dose of 50 Gy (range 44–70 Gy) at 1.8 to 2.0 Gy per fraction. The overall response rate was achieved in 64.8% of patients with complete response in 20.4% of patients at 3 months after completion of IMRT. The median OS was 20.2 months (95% CI = 8.6–31.9), and the actuarial 1-, 2-, and 3-year OS rates were 84.6%, 49.7%, and 36.7%, respectively. The median PFS was 10.5 months (95% CI = 7.3–13.7) and the 1-, 2-, and 3-year PFS rates were 44.2%, 23.4%, and 14.6%, respectively. The responders had a significantly higher OS rate than the nonresponders (3-year OS 48.0% vs 14.4%, *P* = 0.001). During and the first month following IMRT, 10 (18.5%) patients developed grade 3 hematological toxicity, and 3 (5.6%) developed grade 3 hepatic toxicity. No patient experienced grade 4 or 5 toxicity. Radiation-induced liver disease was not observed.

Our findings suggest that IMRT following TACE could be a favorable treatment option for both its safety profile and clinical benefit in patients with unresectable HCC.

## INTRODUCTION

Liver cancer is the fifth most common cancer and the third leading cause of cancer-related deaths worldwide.^1^ Half of the cases and deaths are estimated to occur in China.^2^ It is the fourth most frequently diagnosed cancer and the second leading cause of cancer mortality in China, where it accounts for 355,595 cases (10.54%) of all cancer, and causes 322,417 deaths annually.^3^ Hepatocellular carcinoma (HCC) represents the major histological subtype of primary liver cancers, accounting for 70% to 85% of the total liver cancer burden worldwide.^4^

In general, the tumor stage and underlying liver function are both used to determine the treatment strategy for HCC patients, who are often diagnosed with advanced-stage tumors and underlying chronic liver disease.^5,6^ Complete surgical resection and hepatic transplantation are considered as curative therapy for HCC, but <15% of patients with HCC are indicated for curative surgery.^7,8^ Transarterial chemoembolization (TACE) is one of the most widely used nonsurgical treatments for HCC and has been shown to improve survival compared with symptomatic therapy alone in 2 randomized trials.^9,10^ However, TACE cannot overcome several limitations involving incomplete necrosis owing to dual blood supply around the capsule, multiple collateral circulation or recanalization, and should be complemented by additional treatment modalities.^11^

Although HCC has been reported as a radiosensitive cancer in clinical investigations,^12^ the role of radiotherapy (RT) was limited due to poor tolerance of the whole liver to radiation.^13^ In the advent of 3-dimensional planning systems, 3-dimensional conformal radiotherapy (3D-CRT) allowed the ability to minimize irradiation of normal tissue, and therefore facilitate escalation of the RT dose.^14^ Combination treatment of RT and TACE has been beneficial in patients with unresectable HCC.^12,15–22^ A systemic review and meta-analysis of 17 trials involving 1476 patients, mostly treated with 3D-CRT, found that patients treated with RT following TACE had improved survival compared to patients treated with TACE alone.^14^ A prospective phase 2 multicenter trial found that 3D-CRT following incomplete TACE is a safe and practical treatment option for patients with unresectable HCC.^23^

Intensity-modulated radiotherapy (IMRT), an advanced form of 3D-CRT, can deliver a higher dose distribution that conform closely to the 3-dimensional shape of the target volume while delivering a relative lower dose to the normal liver and/or reducing the dose to other normal tissues.^24–26^ However, there have been few clinical reports on the use of combination treatment of IMRT and TACE for these patients. The aim of this study is to assess the efficacy and toxicity regarding the use of IMRT following TACE for unresectable HCC.

## MATERIALS AND METHODS

### Patient Selection

A retrospective study was conducted using data from the Cancer Hospital of the Chinese Academy of Medical Science. The inclusion criteria for the study were as follows: (1) age ≥ 18 years old; (2) Karnofsky performance score ≥ 70; (3) an initial diagnosis of primary HCC based on biopsy and/or imaging techniques^27^; (4) Child–Pugh class A disease; (5) unresectable tumor status; and (6) a leukocyte count of ≥ 3000/mL; absolute neutrophil count ≥ 1500/mL; hemoglobin level ≥ 90 g/L; platelets ≥ 80 × 10^9^/L; aspartate and alanine aminotransferase levels < 2.5 times the upper normal limit; bilirubin levels < 2 times the upper normal limit; and a prothrombin time-international normalized ratio < 1.5 except if patients were on oral anticoagulation. Exclusion criteria were: (1) extrahepatic metastases (not including regional lymph node involvement); (2) previous abdominal RT. All patients provided written informed consent in accordance with the Declaration of Helsinki. This study was approved by the Independent Ethics Committee of Cancer Hospital, Chinese Academy of Medical Sciences.

### Treatment

#### TACE

TACE was performed by infusion of a mixture of iodized oil contrast medium (Lipiodol; Laboratoire Andre Guerbet, Aulnay-sous-Bois, France) and doxorubicin or cisplatin, which was followed by gelatin sponge particle (Gelform; Upjohn, Kalamazoo, MI) embolization with a 5-F RH catheter (Cook, Bloomington, IN) or Cobra catheter (Cook, Bloomington, IN) or microcatheter (Renegade, Boston Scientific, Natick, MA; Progreat, Terumo, Tokyo, Japan) as selectively as possible through the lobar, segmental, or subsegmental arteries, depending on the tumor distribution and hepatic function reserve. The dosage of lipiodol and doxorubicin or cisplatin was determined by tumor size, vascularity, presence of arterioportal shunt, and underlying liver function. TACE was repeated at intervals of 4 to 6 weeks if it produced a response.

### Radiotherapy

IMRT-based treatment delivery was performed 4 to 6 weeks following TACE. Logistics of treatment planning and treatment delivery have been described previously.^28^ In brief, CT scan (Brilliance 16, Philips Medical Systems, Cleveland, OH) was performed with the patient in a supine position, along with thermoplastic mask immobilization to reduce setup uncertainty and restrain liver motion caused by abdominal breathing. MRI scans were used to optimize target and normal structure delineation using the Pinnacle^3^ 9.0 treatment planning systems (Philips Healthcare, Andover, MA).

The gross tumor volume (GTV) was contoured on intravenous contrast-enhanced scans, as determined by diagnostic dynamic enhanced CT or MRI, including enhanced tumor areas, complete tumor areas filled by the lipiodol-doxorubicin or -cisplatin mixture, tumor areas reflecting complete tissue necrosis after TACE and tumor thrombosis. The clinical target volume (CTV) was defined as the GTV with a surrounding margin of 0.5 cm.^29^ To account for respiratory liver motion and set-up variations, the CTV was expanded by 0.5 cm in the anterior–posterior and medial–lateral directions, and by 1.0 cm in the craniocaudal direction to form the planning target volume (PTV). The whole liver, normal liver which was defined as the total liver volume minus the GTV, spinal cord, small intestine, colon, stomach, and both kidneys were delineated and 3-dimensionally reconstructed. A minimal number of radiation fields, generally 3 to 5 fields of 6 MV photons, and reasonable radiation beam direction were chosen during IMRT planning to ensure that the PTV was covered by the 95% isodose envelope and reduce the doses and volume of normal liver irradiated with the step-and-shoot technique on Elekta Synergy Linac (Elekta, Stockholm, Sweden).

For planning objectives, normal liver received a mean dose of ≤ 28 Gy.^30^ The maximum allowable point dose to the stomach and intestine was set to ≤ 54 Gy, with the volume of organ receiving > 45 Gy be < 15%. The maximum allowable dose of cord should be <45 Gy. The kidney volume receiving a dose ≥ 20 Gy (*V*_20_) was < 50%. In clinical practice, the prescription dose to 95% PTV should be ≥ 50 Gy given in conventional fractionation, 5 days per week; however, the final prescription dose was determined according to dose constraints for organs at risk. Cone beam computed tomography was commonly used for online repositioning prior to treatment.

### Follow-up and Toxicity Assessment

Patients were regularly followed up and received periodic assessments, including serum alpha-fetoprotein, liver biochemistry, routine blood and coagulation tests, chest radiography, and computed tomography and/or magnetic resonance imaging of the abdomen, every 3 months during the first 2 years, and every 6 months thereafter. Patients were followed until death or the censoring date (January 2015).

Toxicities were scored according to the Common Terminology Criteria of Adverse Events (CTCAE), version 3.0. Acute toxicity was evaluated weekly during and the first month following IMRT. Late toxicity was defined as morbidity occurring at least 1 month after the completion of radiotherapy. Patients were evaluated for evidence of radiation-induced liver disease (RILD) 4 months after radiotherapy. RILD was defined as either anicteric elevation of alkaline phosphatase level of at least 2-fold and nonmalignant ascites (classic RILD),^31^ or elevated transaminases of at least 5-fold the upper limit of normal or of pretreatment level (nonclassic RILD),^32^ in the absence of documented progressive disease.

### Evaluations

Tumor response was based on the measurement of the longest diameter of the viable tumor observed on dynamic liver CT or MRI scans obtained 3 months after completion of IMRT according to modified response evaluation criteria in solid tumors (mRECIST) criteria.^33^ In the assessment of in-field (target lesion) response, complete response (CR) was defined as the disappearance of all intratumoral arterial enhancement in all target lesions; partial response (PR) was defined as a decrease of at least 30% in the sum of the diameters of the viable (enhancement in the arterial phase) target lesions, reflecting partial tissue necrosis; progressive disease (PD) was defined as an increase of 20% in the sum of the diameters of viable (enhancing) target lesions or the appearance of any new malignant in-field lesions; and stable disease (SD) was defined as a tumor response between PR and PD. Responders were defined as patients with CR or PR, whereas nonresponders were patients with SD or PD. Overall response was based on the combined assessment of target lesions, nontarget lesions, and new lesions.^33^

### Statistical Analysis

Statistical analysis was performed on follow-up data that were collected up to May 2015. Overall survival (OS) was calculated as the number of months from the date of IMRT delivery to the date of death from any cause or the last follow-up. Progression-free survival (PFS) was calculated from the date of IMRT delivery to the date of disease progression, relapse, death related to disease, or the last contact. Infield-failure-free survival (IFFS) was defined as the number of months from the start of RT to the date of infield failure occurring at any time or the last follow-up. Outfield-failure-free survival (OFFS) was estimated as the number of months from the start of RT to the date of first outfield failure or the last follow-up, independent of the presence or absence of infield failure. Kaplan–Meier survival analyses were used to calculate actuarial survival and local control rate. Univariate analysis was performed on potential prognostic factors using the log-rank test. Statistical significance was defined as *P* < 0.05. All statistical analyses were performed using SPSS software, version 17 (SPSS, Chicago, IL).

## RESULTS

### Patient Characteristics

Between Jan 2009 and Jun 2014, 54 patients with unresectable HCC received IMRT following TACE at our institution were reviewed (Table 1). Fifty-one men and 3 women were enrolled. The median patient age was 53 years (range, 34–82). There were 26 patients (48.1%) with tumor thrombosis, 11 patients (20.4%) with tumor larger than 10 cm, and 4 patients (7.4%) with local regional lymph node metastases. There were 4 Stage I (UICC 7th) patients who were not suitable for surgery: 2 patients age older than 70, and 2 patients with tumor larger than 9 cm. All patients underwent median 3 cycles (range, 1–6) of TACE.

**TABLE 1:**
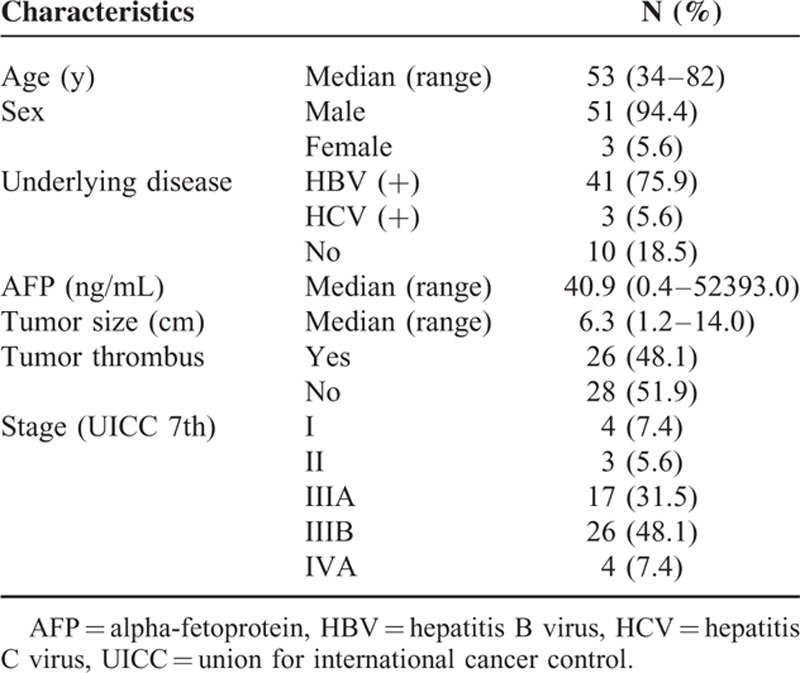
Patients Characteristics (n = 54)

### IMRT Parameters

Fifty-four patients were treated with IMRT to their 64 respective lesions. The median RT dose of 95% PTV was 50.0 Gy (range, 44.0–70.0 Gy). The mean and standard deviation (SD) of GTV and PTV were 309.3 ± 376.3 mL and 596.5 ± 536.4 mL, respectively. And the mean normal liver volume was 1235.3 mL (range, 720.7–2221.3 mL). The mean *D*_mean_ of normal liver was 21.5 Gy (range, 12.4–28.0 Gy), and the average *V*_10_, *V*_20_, *V*_30_, *V*_40_ of normal liver were 54.9%, 40.1%, 30.6%, 23.6%, respectively. The dose–volume histogram (DVH) analysis for organs at risk is listed in Table 2.

**TABLE 2:**
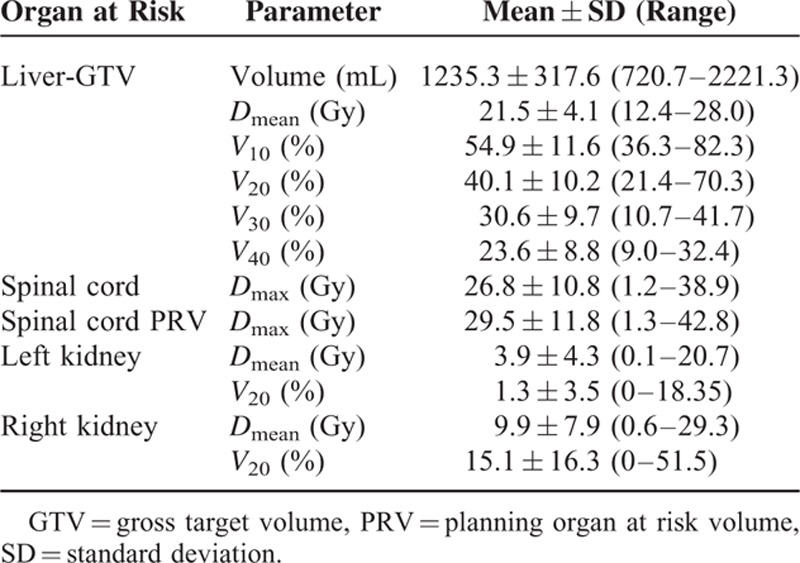
Summary of the Dose–Volume Histogram Analysis for the Organs at Risk

### Tumor Response

Three months after the completion of IMRT, an objective response was observed in all patients. There was in-field (target lesion) CR in 11 patients (20.4%), PR in 29 patients (53.7%), SD in 8 patients (14.8%), and PD in 6 patients (11.1%). The overall CR, PR, SD, PD were observed in 11 (20.4%), 24 (44.4%), 9 (16.7%), and 10 (18.5%) patients, respectively. The objective in-field and overall response rates were 74.1% and 64.8%, respectively.

### Patterns of Failure

The median follow-up period for all patients was 28.7 months (range, 7.0–72.3 months). A total of 41 failures (75.9%) were noted. Local progression within the RT field occurred in 13 patients (24.2%). Intrahepatic metastasis or new lesions out of the RT field developed in 25 patients (46.3%), and extrahepatic failure (distant metastasis) was found in 14 patients (26.0%).

### Survival Outcomes

**FIGURE 1:**
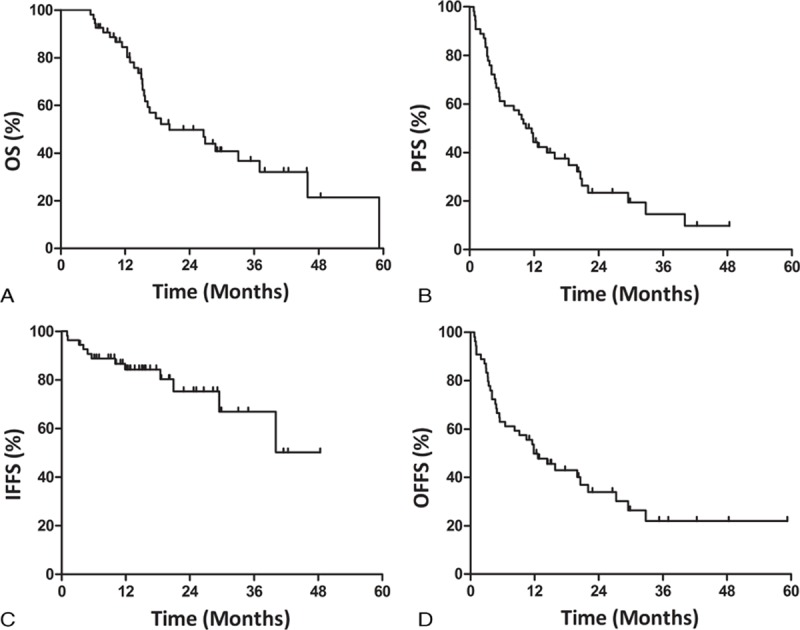
(A) Overall survival (OS), (B) progression-free survival (PFS), (C) infield-failure-free survival (IFFS), and (D) outfield-failure-free survival (OFFS) of all patients (n = 54). IFFS = infield-failure-free survival, OFFS = outfield-failure-free survival, OS = overall survival, PFS = progression-free survival.

Two patients were lost to follow-up at 7 months and 10 months, respectively. At the time of analysis, 30 of 54 patients (55.6%) had died. The median OS was 20.2 months (95% CI = 8.6–31.9) and the actuarial 1-, 2-, and 3-year OS rates were 84.6%, 49.7%, and 36.7%, respectively (Figure 1A). The median PFS was 10.5 months (95% CI = 7.3–13.7) and the 1-, 2-, and 3-year PFS rates were 44.2%, 23.4%, and 14.6%, respectively (Figure 1B). The median IFFS was not achieved. The 1-, 2-, and 3-year IFFS rates were 84.3%, 75.2%, and 66.9%, respectively (Figure 1C). The median OFFS was 11.9 months (95% CI = 6.2–17.5) and the 1-, 2-, 3-year OFFS rates were 49.8%, 33.9%, and 22.0%, respectively (Figure 1D). In responders, median OS was 33.0 months (95% CI = 20.9–45.2) and the 1-, 2-, and 3-year OS rates were 94.2%, 63.0%, and 48.0%, respectively. In nonresponders, the median overall survival duration was 13.6 months (95% CI = 11.4–15.9) and the 1-, 2-, and 3-year OS rates were 64.8%, 21.6%, and 14.4%, respectively. The responders had a significantly higher overall survival rate than the nonresponders (*P* = 0.001). In univariate analysis, AFP (*P* = 0.041), tumor size (*P* = 0.002), and response to treatment (*P* = 0.001) were significantly associated with OS; and AFP (*P* = 0.013), tumor size (*P* = 0.000), tumor thrombus (*P* = 0.018), and response to treatment *(P* = 0.005) were significantly associated with PFS.

### Toxicity

The observed radiation-related acute toxicities are summarized in Table 3. Three patients (5.6%) experienced grade 3 hepatic toxicity, and 10 (18.5%) suffered from grade 3 hematologic toxicity. All patients recovered from the acute toxicities after 1 to 4 weeks of treatment, and no patient had any interruption of irradiation. No patients experienced ≥ grade 4 hepatic and hematologic toxicity. Moreover, no patient developed classic or nonclassic RILD, gastroduodenal ulcer or perforation, and other severe late toxicities. Only 4 patients experienced grade 2 gastritis or duodenitis.

**TABLE 3:**
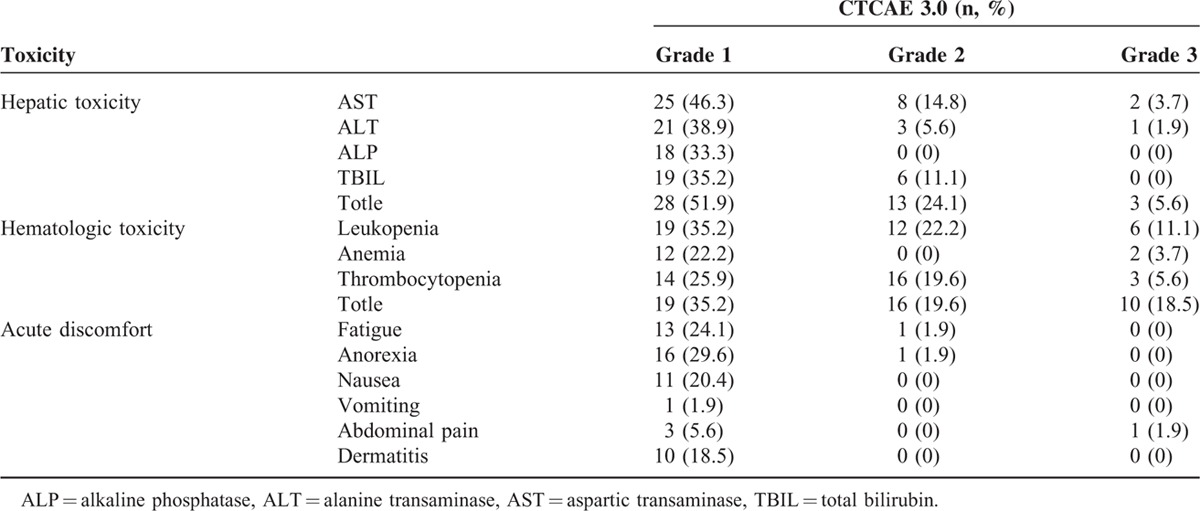
Acute Radiation-Related Toxicities

## DISCUSSION

Most HCC patients are not surgical candidates due to tumor extent or compromised hepatic function. TACE has become the most popular treatment modality for unresectable HCC patients; however, the reported response rates and survival benefits of this modality were poor. A meta-analysis of TACE for unresectable HCC showed that TACE induced objective responses in 35% of patients (range, 16%–61%).^34^ Even in an encapsulated tumor, which is favorable tumor type for TACE, the necrosis rate was reported to be <44%.^35^ RT may eradicate tumor cells at the periphery of the primary HCC that are maintained through the dual blood supply and the supply from collateral circulation or recanalization of the embolized artery after TACE.^36^ A meta-analysis reported that patients receiving TACE plus RT showed significantly better 1-year survival [OR, 1.36 (95% CI = 1.19–1.54)] and CR [OR, 2.73 (95% CI = 1.95–3.81)] compared with TACE alone; survival benefit also progressively increased for 2-, 3-, 4-, and 5-year survival.^37^

Numerous clinical studies have demonstrated the superiority of 3D-CRT plus TACE over TACE alone in unresectable HCC patients, with response rates ranging from 18% to 91.1%, CR rates ranging from 0 to 20.9%, and median survival time ranging from 10.6 to 23.5 months (Table 4).^18,20–23,38–40^. In the present study, IMRT, a more sophisticated form of 3D-CRT, following TACE has been performed in all patients, of which the objective overall response rate (64.8%), CR rate (20.4%), and median survival time (20.2 months) were comparable with the results of previous studies.^19,21–24,38–40^ Thus, combined local IMRT with TACE may be a promising therapeutic approach for unresectable HCC.

**TABLE 4:**
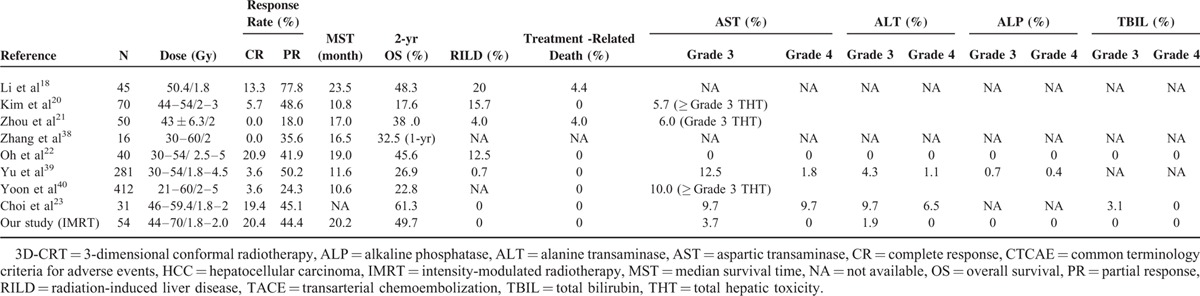
Comparison of Efficacy and Toxicity Between 3D-CRT and IMRT (Our Study) Following TACE for Unresectable HCC

Unresectable HCC patients referred for RT usually have underlying liver disease, and clinicians should consider not only tumor control but RT-related complications as well. Radiation-induced hepatic toxicity has been a major limitation of RT for HCC.^41^ The incidence of RILD and treatment-related death in 3D-CRT following TACE have been shown to range between 0%–20% and 0%–4.4%, respectively (Table 4). Yu et al^39^ reported that grade 3 AST, ALT, and ALP elevation during RT were observed in 35 (12.5%), 12 (4.3%), and 2 (0.7%) patients, and the grade 4 AST, ALT, and ALP elevation in 5 (1.8%), 3 (1.1%), and 1 (0.4%) patients, respectively. After RT, grade 3 or 4 clinical hepatic dysfunction was observed in 15 patients (5.3%).^39^ Choi et al^23^ reported that grade 3 of AST, ALT, and bilirubin elevation during and after RT were observed in 3 (9.7%), 3 (9.7%), and 1 (3.2%) patients, meanwhile 3 (9.7%) and 2 (6.5%) patients suffered from grade 4 AST and ALT elevation. In the present study, there were no incidences of RILD, treatment-related liver failure or death; only 3 patients (5.6%) experienced grade 3 hepatic toxicity, with no occurrence of ≥ grade 4 toxicities. Overall, the treatment regimen presented in the present study produces similar efficacy and offers a more favorable safety profile than current comparable treatment plans.

In contrast to 3D-CRT, IMRT uses an inverse planning algorithm to generate multiple nonuniform-intensity beams that allows for a higher dose conformality to the tumor while reducing the dose to the normal liver and other sensitive structures^24,26^ Kuo et al^26^ demonstrated that the mean dose to the normal liver, which was associated with the risk of RILD,^42^ was significantly lower for IMRT (19.31 ± 2.89 Gy) than for 3D-CRT (21.58 ± 3.01 Gy) (*P* < 0.05). The dose regions of the normal liver were higher for *V*_40_, *V*_30_, and *V*_20_ with 3D-CRT (23.05 ± 4.06%, 32.10 ± 6.80% and 42.12 ± 7.56%) than with IMRT (18.61 ± 4.13%, 26.23 ± 5.87% and 37.16 ± 8.65%) (*P* < 0.01).^26^ The normal tissue complication probability (NTCP) value for 3D-CRT (7.57 ± 4.36) was significantly higher than that for IMRT (3.98 ± 3.00) (*P* < 0.01).^26^ Cheng et al^43^ reported similar findings that demonstrated the diverse dosimetric effect of IMRT on the liver, with a significant reduction in NTCP (23.7% vs. 36.6%, *P* = 0.009).

Novel rotational IMRT modalities used to treat patients with HCC include helical tomotherapy (HT) and volumetric modulated arc therapy (VMAT). Studies have shown that 0% to 5% of patients treated with HT and concurrent chemotherapy developed RILD.^44–49^ Kong et al^47^ observed RILD in 5% of patients treated with HT monotherapy, with similar response rates as the present study. Yoon et al^48^ reported RILD in 2/65 patients (3.1%) treated with HT and concurrent transarterial chemoinfusion; median survival time (20 months) was similar to the present study. Wang et al^50^ described a response rate of 64% in 138 HCC patients treated with VMAT, 34 (25%) of which developed RILD. As HCC is always surrounded by normal liver parenchyma, rotational IMRT modalities may not offer more liver protection than IMRT.^26^ Hsieh et al^51^ reported no significant differences in CTV and PTV coverage between IMRT and HT, and while the 2 techniques delivered similar mean radiation doses to the liver, *V*_10_ was significantly larger in HT than IMRT (*P* < 0.05). Most recently, Song et al^52^ reported that by changing the treatment modality from HT to IMRT, the liver dose and the probability of radiation-induced hepatic toxicity could be reduced without sacrificing the target coverage, especially in patients where a high dose is delivered to the liver. IMRT also achieved a significantly lower mean radiation dose to the normal liver compared to RapidArc (VMAT), where the regions of the normal liver were lower for *V*_20_ and *V*_10_ in IMRT than in VMAT.^26^

As a single institution experience, the generalization of our results is limited. The retrospective design and small population size may result in unforeseen biases. The effect of these drawbacks on outcome is not known; the results should be prospectively validated in a larger study.

In conclusion, our findings suggest that IMRT following TACE could be a favorable treatment option for both its safety profile and clinical benefit in patients with unresectable HCC. This provides support to conduct prospective and randomized trials to further determine the role of this treatment strategy.
